# Cyclodextrins as Multi-Functional Ingredients in Dentistry

**DOI:** 10.3390/pharmaceutics15092251

**Published:** 2023-08-31

**Authors:** Susana Santos Braga

**Affiliations:** LAQV-REQUIMTE, Department of Chemistry, University of Aveiro, 3810-193 Aveiro, Portugal; sbraga@ua.pt

**Keywords:** cyclodextrins, drug delivery, oral hygiene, dental repair, enamel, osteogenesis

## Abstract

Cyclodextrins are present in a variety of oral hygiene compositions. The present work describes the role of cyclodextrins in several toothpastes and mouthwashes that are already available in the market, as well as their prospective use in other applications as investigated in studies in the literature. Moreover, cyclodextrins are under study for the development of materials used in various techniques of dental repair, such as fillings, cements and binders therein. Their role in each of the innovative materials is presented. Finally, the prospect of the use of cyclodextrin-based delivery systems for the oral cavity is introduced, with a focus on new cyclodextrin molecules with dual action as bone-targeting agents and osteogenic drugs, and on new cross-linked cyclodextrin particles with a high drug loading and sustained drug delivery profile for the treatment of diseases that require prolonged action, such as periodontitis. In conclusion, cyclodextrins are herein demonstrated to act as versatile and multi-action ingredients with a broad range of applications in dentistry.

## 1. Introduction

The oral cavity is a complex and biodiverse small-scale ecosystem that hosts a vast community of microorganisms, from bacteria and protozoa to fungi and even viruses [1,2]. The intricate interplay between the different species of the oral microbiome has a crucial role in maintaining oral health. Their delicate balance can be easily disrupted by a variety of factors and, when this happens, it can lead to a variety of local diseases, such as cavities, gum disease, and halitosis, as well as some systemic diseases. Diet is an important factor affecting the oral microbiome, with the excess intake of processed foods, a common action in modern developed societies, being associated with a higher incidence of cavities and periodontal disease; the reduced intake of fruits and vegetables, causing less abundance of vitamins in the human host, also contributes to this scenario [3,4]. In this context, maintenance of adequate oral hygiene practices is essential to counteract the buildup of acid compounds resulting from sugar fermentation by the microbiome, avoid biofilm formation and reduce the risk of oral diseases [4].

**Figure 1:**
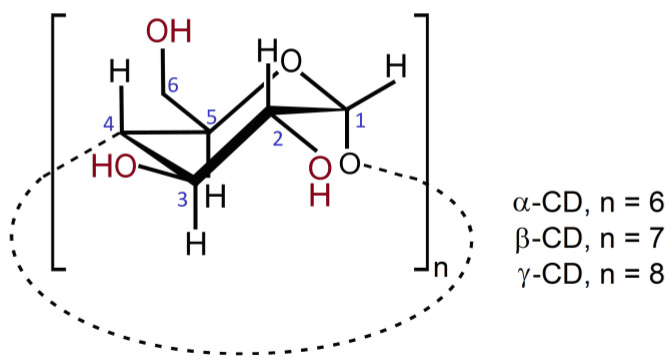
Chemical structure and atom labelling of native cyclodextrins. Hydroxyl groups, the anchoring points for chemical modification to introduce functional groups, are depicted in dark red.

Consumers are increasingly looking for eco-friendly options when it comes to their oral care routine, driven by increased environmental awareness and concerns about ingredients in traditional oral care products [5]. A well-known example is triclosan, a ubiquitous disinfecting agent in mouthwashes, toothpastes and other consumer care products that is toxic to aquatic species [6]. Addressing these concerns and market demands, companies are developing oral care compositions based on natural and organic ingredients, which, besides bringing benefits to the environment, also contribute to a healthier lifestyle for the product end-users. As such, the use of cyclodextrins, multi-faceted compounds that can act as solubilisers [7,8,9,10,11], activity enhancers [9,12,13], taste-masking agents [14,15] and shelf-life extending agents [10,16], emerged as a logical choice. Cyclodextrins are cyclic oligosaccharides that can be obtained from starch by natural bacterial degradation or by enzymatic digestion. These compounds can have a varied number of glucose units, the most abundant ones being those with six (α-CD), seven (β-CD) or eight (γ-CD) D-glucose units that receive, for their presence in nature, the denomination of native cyclodextrins (Figure 1). The glucose units are linked together by α-1,4 glycosidic bonds, affording cyclodextrins with a quite unique molecular geometry in the shape of a truncated cone. Notably, the secondary hydroxyl groups of the glucose residues are directed towards the wider rim of the cone, and the primary hydroxyls, bound to C6, face the narrower rim, which overall conveys them with good aqueous solubility. In turn, the inner cavity, lined with protons, is sufficiently hydrophobic to hold apolar guests of adequate shape and size, rendering them more water soluble. The resulting adducts are called inclusion compounds, and they are found in numerous applications of the pharmaceutical industry, from solid oral dosage forms to injectables [17,18,19], and in products of the cosmetics and personal care branch [20,21]. Formation of inclusion compounds with cyclodextrins is a well-known strategy for improving the solubility of various active pharmaceutical ingredients that can be found in several commercial solid dosage forms, such as the birth control pill ‘Yaz^®^’ [22] and tablets of painkillers, anti-acids and anti-histamine agents [18]. In the case of painkillers, the solubilising effect of cyclodextrins helps achieve a faster onset of action, which is useful, for instance, in the quick control of pain following dental surgery [23]. Moreover, cyclodextrins offer multiple advantages when they are used to encapsulate antibiotics, bringing improved solubility, masking their unpleasant taste [14] and, in several instances, increasing antimicrobial potency in the mouth cavity [24].

Chemical modification of cyclodextrins through the introduction of functional groups at their hydroxyl residues affords a large variety of derivatives with tailored physico-chemical properties. Thousands of cyclodextrin derivatives have already been synthesised and reported [25] but, of these, only a few are suited for use in humans, the most common ones being 2-hydroxypropylated (HP) derivatives and methylated derivatives (see Table 1 for pharmaceutical products containing cyclodextrins and their corresponding routes of administration). Some of these have restricted use in what concerns ingestion due to concerns with renal effect and haematological effects, and even the native cyclodextrins have some restrictions to use [19]. Noteworthy restrictions include the oral daily intake limit for β-CD, which cannot surpass 5 mg per kg of body weight, and daily oral/parenteral intake limits of 200 mg per kg for the remaining cyclodextrins [19]. The methylated derivative known as RAMEB because of the random position of its methyl groups (averaging 1.8 methoxy groups per glucose unit) cannot be used systemically. RAMEB has a strong haemolytic activity [26,27] and renal toxicity superior to that of the parent β-CD, being approved for nasal administration at concentrations up to 10% [19].

When considering administration to the oral cavity, that is, the preparation of formulations that are not meant to be swallowed, cyclodextrin derivatives can be employed with greater degrees of freedom. HPβCD is most probably the one with the highest use for its high safety and tolerability. Regarding RAMEB, its safety of use in the oral cavity was evaluated on human oral epithelial cell cultures (TR 146 cell line), with no significant effects on cell viability at concentrations up to 5%; in turn, the concentration of 10% was shown to be cytotoxic and pro-inflammatory, inducing interleukin-1 expression; formulations for the oral cavity that contain RAMEB should thus ensure that its concentration does not surpass 5% [28].

## 2. Cyclodextrins in Oral Hygiene Compositions

Cyclodextrins are useful to oral care compositions in a variety of ways: they help solubilise apolar ingredients, they make the included substances more compatible with the remaining components of the formulation, they increase the bioavailability of active ingredients and, by doing so, they contribute to reducing the concentration of actives in the final product while maintaining the efficacy.

### 2.1. Toothpastes

Triclosan is a common antimicrobial and antiseptic agent in toothpaste compositions. In a small-scale trial with human volunteers, triclosan was demonstrated to have increased bioavailability and efficacy when released from toothpaste compositions that were added with β-CD [29]. Moreover, cyclodextrin inclusion compounds with triclosan are present in commercial compositions: in the toothpaste Bexident^®^ Gums, triclosan is present both in the free form and in the cyclodextrin-included form as a way to control the proliferation of the mouth microflora and protect the gums of gingivitis-prone patients [30].

Interestingly, cyclodextrins themselves can act as medicinal agents and help control bacterial proliferation. An example is the toothpaste G·U·M WellPlus^®^, developed against gingivitis and periodontitis, which contains β-CD as a “dispersing ingredient”. The role of β-CD is defined as that of a dispersing agent, not in the conventional form (which would be to homogenise the ingredients of the composition), and rather to help disperse the build-up of bacteria that causes periodontal diseases; this way, β-CD is claimed to block bacterial growth and sterilise periodontal bacteria before they colonise the tooth-gum interface and the spaces between the teeth [31].

A variety of other cyclodextrin-containing toothpastes is available. In the following compositions, the use of cyclodextrins is privileged for their natural origin as the toothpastes contain preferentially natural ingredients. An example is the whitening toothpaste Blanx Pro Pure White from the Italian company Coswell S.P.A., based on cyclodextrins, enzymes and plant extracts [32,33]. The composition relies on the component usnic acid, present in the Arctic lichen extract, for an antibacterial and anti-plaque effect; the enzyme components, papain and bromelain, bring antibacterial and stain-removing effects; finally, mild abrasion is ensured by the presence of fine-grained silica. Note that the type of cyclodextrin used in this composition is not disclosed [33]. Developed by Nature’s Gate, the Organic Whitening Toothpaste is another example of a composition containing unspecified cyclodextrins in tandem with a variety of plant extracts such as green tea, fig, bamboo, licorice and tea tree oil; whitening agents in this composition include sodium bicarbonate and silica particles [34]. Another example is the Thailandese toothpaste named Dentiste’s Herbapeutic Toothpaste, available worldwide from online suppliers [35]. This composition is based on cyclodextrin complexes of fourteen plant extracts (once again, included in a non-specified cyclodextrin). It further contains xylitol, vitamin C and micronised silica. It is designed to whiten teeth and control halitosis from the first night of use by inhibiting nighttime oral bacteria, which grow rapidly in the mouth cavity during sleep. Its action is claimed to result from the release of antimicrobial plant oils by the cyclodextrin complexes, followed by the inclusion and entrapment of malodorous compounds by the free cyclodextrin molecules. An antimicrobial dental composition (e.g., paste, gel) based on cyclodextrin inclusion compounds of persimmon juice or grapefruit essential oil is patented in Germany [36]. The cyclodextrins to be used include α-CD, β-CD, γ-CD or a branched cyclodextrin derivative in which one to four glucose molecules are α-1-6 linked to these cyclodextrins, and they are used in order to facilitate formulation by turning the persimmon juice and grapefruit essential oil guests into powders that are subsequently incorporated into the formulation.

A toothpaste with the inclusion compound of β-CD·coenzyme Q10 is reported to help control mild to moderate gingivitis [37]. This study is based on the premise that the development and progression of periodontal diseases are related to a deficiency of coenzyme Q10, a lipid-soluble antioxidant essential for the maintenance of cellular energy production and the health of human cells. It comprises a test population of twenty-four volunteers with mild to moderate gingivitis, divided into two groups, one using non-supplemented toothpaste (control) and the other using the same brand of toothpaste supplemented with 1.5% of the inclusion compound β-CD·coenzyme Q10, brushing twice daily with their assigned toothpaste composition for a period of eight weeks. The experimental group showed positive developments upon eight weeks of using the supplemented toothpaste, with 66% of subjects having reduced gingival versus 33% in the control group. These results are further confirmed by measurement of salivary immunoglobulin (sIgA) using an immunoassay kit, results being considered positive for a 20% or more reduction in the levels of sIgA; again, 66.6% of the experimental group showed improvement by this standard, while 33.3% of the control group showed improvement, but this did not reach statistical significance. In conclusion, the toothpaste composition with the inclusion compound β-CD·coenzyme Q10 significantly reduced moderate gingivitis.

### 2.2. Toothpaste Additives

The company Highsmile has recently released a powder that is intended to be added to any regular toothpaste and turn it into a teeth-whitening composition. The product, named PAP Whitening Powder, uses phthalimidoperoxycaproic acid as the whitening agent and nano-hydroxyapatite for further abrasive power; it further comprises potassium citrate, a desensitising agent that helps to relieve pre-existing sensitivity, as well as mint aroma and β-CD [38].

### 2.3. Mouthwashes

The company Johnson and Johnson holds a patent on the use of cyclodextrins as solubilisers and stabilisers for phenolic antimicrobial compounds in mouthwash compositions [39]. The compounds comprise menthol, eucalyptol, methyl salicylate, thymol, triclosan and mixtures thereof. The phenolic compounds solubilised by cyclodextrins have improved bioavailability and excellent low-temperature stability. The patented compositions are claimed to help retard the development of plaque, treat gingivitis, and treat the presence of microorganisms in the oral cavity.

**Figure 2:**
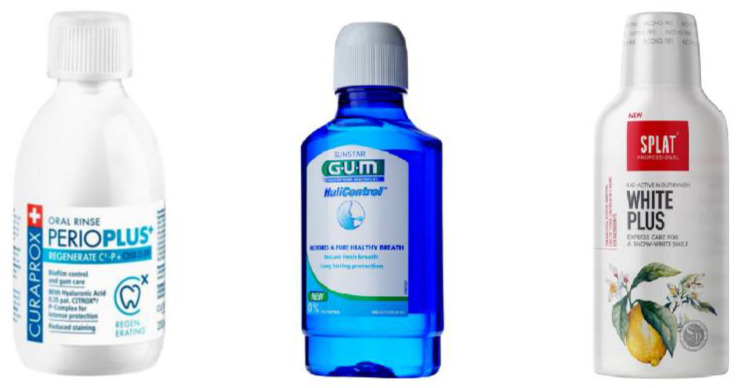
Commercially available mouthwash compositions containing cyclodextrins.

A few cyclodextrin-containing mouthwash compositions are available in the market (Figure 2) [40,41,42]. Similarly to the toothpaste compositions, the exact type of cyclodextrin used is not specified on the list of ingredients.

The composition G·U·M Halicontrol^®^ is designed to prevent halitosis by using ingredients that are able to sequester and neutralise the molecules that cause bad odour in the mouth [40], a role that can be attributed to the presence of cyclodextrin. The composition further contains cetylpyridinium chloride for biocidal action that controls mouth bacteria proliferation.

The mouthwash composition named Curaprox Perio Plus Regenerate combines cyclodextrins with two disinfecting ingredients: chlorhexidine, a biocide of widespread use in dental hygiene products, and an anti-germ component of natural origin, Citrox^®^, which is a mixture of bioflavonoids from bitter orange, *Citrus aurantium amara* [41]. The antimicrobial action of Citrox^®^ is quite strong and it has a broad spectrum, as demonstrated by in vitro studies [43,44]. Moreover, Perio Plus has the ability to reduce, after being used in mouth rinsing, the viral load in saliva, as demonstrated by a clinical trial with 196 volunteers (infected with SARS-CoV-2, 88 in the treatment group and 88 in the placebo group) [45]. To better understand the role of cyclodextrins in Perio Plus, it is important to note that the main components of Citrox^®^ are the flavonoids neohesperidin and naringin [46,47], two compounds with a strong bitterness and the main taste components in bitter oranges. Cyclodextrin is used to include, stabilise and mask the bitter taste of the flavonoids, ensuring, as a result of the inclusion, that the product is well accepted. As stated by the manufacturer, “Perio Plus does not have any impact on the sense of taste” of the users [41].

The composition with the name SPLAT^®^ White Plus is another example of CD-containing mouthwash designed to have only natural ingredients [42]. It contains a combination of Japanese licorice tree extract and lactic proteins with the purpose of preventing cavities and dental plaque; the whitening action is based on pineapple extract, rich in bromelain that not only gently whitens teeth but also promotes oral cavity immunity. It further contains the lysate of bifidae ferments, claimed to promote cleansing, as well as a lemon essential oil to freshen the breath, thyme essential oil to strengthen soft oral tissue and zinc salts to eliminate bad odours and provide fresh breath.

## 3. Cyclodextrins in Dental Repair

Cyclodextrin inclusion compounds, cyclodextrin-based materials and cyclodextrin conjugates are under investigation for different stages of repair of tooth cavities and mouth lesions. These applications can be as simple as the formation of inclusion compounds with drugs or molecules of anaesthetics used in surgery, or more elaborate, such as the development of novel materials with cyclodextrins to act as dental cement or in the control of the periodontal disease. Cyclodextrins are further employed in innovative osteogenic strategies.

### 3.1. Cyclodextrin Inclusion Compounds with Anesthetics

Articaine, a widely used local anesthetics in dentistry, was shown to benefit from inclusion into cyclodextrins regarding safety: a 1:1 inclusion compound of HPβCD and articaine, prepared by co-dissolution/freeze-drying, had lower in vitro cytotoxicity of the drug against human primary gingival fibroblasts (HGF cells) than the free drug, with IC_50_ values increasing from 16 μM (pure articain) to >20 μM, the highest concentration tested, with HPβCD·articaine [48]. Moreover, fluorescence microscopy of the gingival cells revealed loss of cytoplasm volume and the presence of apoptotic bodies on cells treated with 10 and 20 μM of pure articaine, which were not observed with HPβCD·articaine at the same concentrations.

Cyclodextrin’s inclusion of anaesthetic drugs, such as bupivacaine [49], ropivacaine [50] and tetracaine [51], was shown to increase the duration and intensity of the sensory blockade effect in various animal studies. However, these studies targeted the spinal cord and sciatic nerve. The positive results, along with the lack of knowledge of whether the efficacy in local dental anaesthesia would be equivalent, prompted evaluation, in a mouse model, of an inclusion compound, HPβCD·bupivacaine, in dental anaesthesia by blocking the inferior mandible alveolar nerve. The inclusion compound was compared with a solution containing bupivacaine + adrenaline, having shown equivalent results in both times of onset and duration of anaesthesia [52]. While not capable of prolonging the length of the anaesthetic effect, HPβCD·bupivacaine may pose as an adrenalin-free alternative for future clinical use in dentistry.

### 3.2. Cyclodextrins in Dental Materials—Fillings, Pastes and Cements

Dental repair commonly uses cement-based materials in a variety of operations, from filling small dentin cavities to more complex scenarios such as resolving perforation of the root canal system and direct pulp capping. A variety of inorganic and composite materials can be used, such as calcium silicate cement (based on the Portland cement) [53], zinc phosphate materials and composites of methacrylate resins with varied fillers (silicon dioxide, boron silicates and, for radio-opacity, small amounts of barium, strontium or zinc) [54].

#### 3.2.1. Materials with α-Cyclodextrin

An adhesive bone paste for dental implants and soft tissue interfaces containing α-cyclodextrin is reported [55]. The paste mainly comprises poly(vinyl alcohol) modified with several nonanyl groups at the alcohol positions (PVA-NA), on which α-CD forms multiple inclusions along the polymer side chains. The α-CD-containing bone paste was tested against two commercial materials, biopex-R (calcium phosphate cement) and Nanohap^TM^ (nano-sized hydroxyapatite), as well as against plain PVA-NA (that is, the α-CD-free version), and it showed the highest bonding and shear adhesion between commercially pure titanium plates and soft tissue like collagen casing. Regarding compressive strength, the α-CD-containing PVA-NA paste reached 14.1 ± 3.8 MPa within 24 h incubation. Further tests comprised in vitro biological evaluation. Mouse fibroblast cells (L929 line) were cultured on 10 mm Ø disks made of the α-CD-containing PVA-NA bone paste to investigate its ability to promote cell adhesion and proliferation. Results obtained at 24 h of incubation showed high cell adhesion on the surfaces of both Biopex-R (positive control) and the cured α-CD-containing PVA-NA bone pastes. In the latter case, adhesion was attributed to the presence of alkyl groups that helped anchor the fibroblasts.

**Figure 3:**
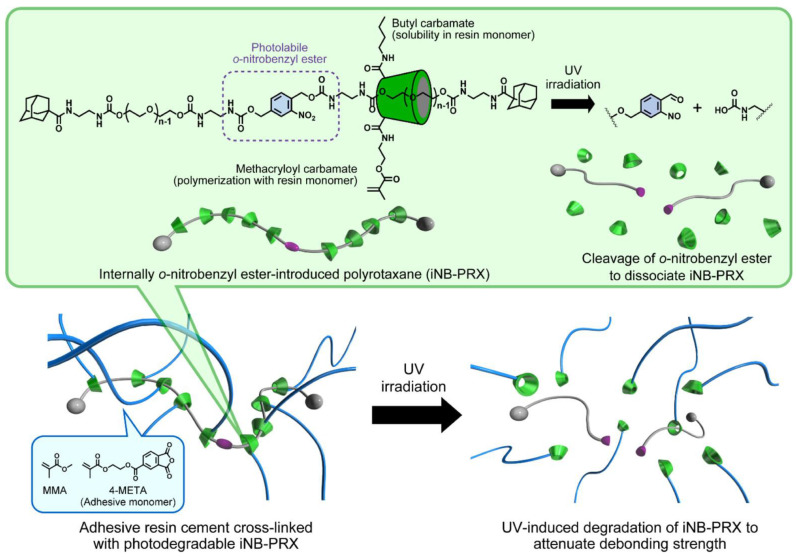
Schematic illustration of internally *o*-nitrobenzyl ester-introduced photodegradable polyrotaxanes (iNB-PRX) and UV-induced embrittlement of adhesive resin cements containing methylmethacrylate (MMA), 4-methacryloxyethyl trimellitate anhydride (4-META), and iNB-PRX. Reproduced from Matsunaga et al. [56] under a Creative Commons Licence.

In some treatments, such as orthodontic brackets, temporary crowns, and temporary splinting of teeth, dental materials are temporarily adhered to the tooth using adhesive resin cements. The subsequent removal of the adhered materials from the tooth surfaces still poses challenges as it is typically carried out by mechanical detachment or destruction of the materials, with the risk of damaging or fracturing the enamel layers. As an alternative, a photodegradable cross-linker agent in dental resin cements based on the use of α-CD is reported [56]. The material, threaded with α-CD molecules, contains a photolabile *o*-nitrobenzyl ester (Figure 3) and it was used as a cross-linker for poly(methyl methacrylate) (PMMA) blocks. Under exposure to UV light, the α-CD-threaded polymer gradually degrades into its components, methacryloyl and butyl carbamate-modified α-CD, with 60% degradation being reached after only 5 min of irradiation. Conversely, the polymer without α-CD (control material) was not significantly changed by UV irradiation.

#### 3.2.2. Materials with β-Cyclodextrin or Its Derivatives

Methacrylated β-CD is an innovative dental monomer that can be used as a filling paste and polymerised in situ to form dental resins; at the same time, the cavity of β-CD retains the ability to include guest molecules, being thus suited for carrying pharmacologically active substances [57]. Regarding the use of methacrylated β-CD in a dental composite, a composition with suitable viscosity is reported to have 75% glass filler (silanated barium oxide-containing glass) and 25% of a resin with equal parts methacrylate and methacrylated β-CD [58]. It must be noted, however, that the use of this new methacrylated β-CD remains merely experimental and that it is not approved in humans.

**Figure 4:**
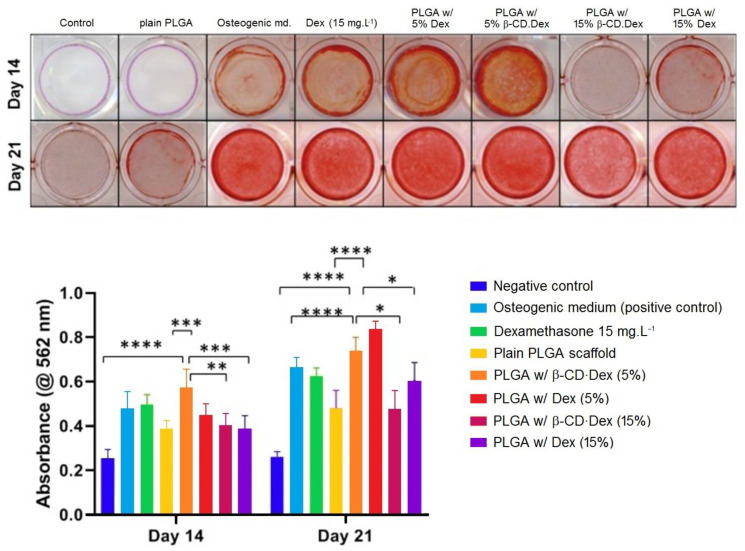
Osteogenic effect of free/β-CD-included dexamethasone. Alizarin red stained wells (top of the figure) show the effect, at day 14, of both pure and β-CD/PLGA scaffold-delivered dexamethasone (Dex) on the induced mineralisation of stem cells from human-extracted deciduous teeth. Quantification of alizarin red S staining (graph at the bottom part of the figure) shows the effect of different concentrations of Dex, as released by the PLGA scaffolds with the pure drug or β-CD·Dex, on the induced matrix mineralisation in the stem cells. Adapted with permission from Daghrery et al. [59]; copyright ©2020 Elsevier.

Repair of caries that reach as deep as the dental pulp is usually conducted with dedicated materials such as calcium hydroxide, Ca[OH]_2_ and mineral trioxide aggregate (MTA). The procedure aims at preserving dental pulp vitality and stimulating tertiary dentin formation through a calcified barrier, which is called “direct pulp capping”. The materials therein employed are, however, not entirely devoiced of complications, with calcium hydroxide causing dentin inflammation and MTA being difficult to manipulate and taking too long to set during the procedures. In the search for alternative, biocompatible materials for pulp capping, a combination of electrospun biodegradable fibres with β-CD·dexamethasone is reported [59]. The inclusion compound, with a stoichiometry of 1:1, is prepared by co-dissolution/freeze-drying and then added, in amounts corresponding to 5, 10 and 15% (wt) of dexamethasone, to poly(lactic-co-glycolic) acid (PLGA) solutions that are subjected to electrospinning. The resulting scaffolds have an excellent in vitro drug release profile, which is sustained over time and lasts, at least, up to 28 days (maximum time of observation). Moreover, cell culture studies show that the scaffolds are biocompatible and osteogenic, inducing the proliferation of stem cells from human-extracted deciduous teeth during days 1 to 3 of incubation, and causing differentiation between days 7 and 14. Osteo/odontoblastic differentiation is assessed by measuring the activity of the alkaline phosphatase (ALP) membrane-associated enzyme that marks the early stages of osteoblastic differentiation. Good ALP activity is observed for the scaffold having the inclusion compound β-CD·dexamethasone at a dose equivalent to 5% of the drug [59], as dexamethasone is known to induce osteogenesis at concentrations up to 100 μM, while, at higher concentrations, dexamethasone has the opposite effect and becomes an osteogenesis inhibitor [60]. Microscopy of the scaffolds after cell colonisation, using alizarin red staining to evidence the accumulation of calcium, shows a clear increase in osteogenic potency of the dexamethasone drug in the scaffolds with the inclusion compound, in contrast with the cells cultured with only the pure drug (Figure 4). Once again, higher concentrations of β-CD·dexamethasone in the scaffold leads to lower calcification after a prolonged culture time due to the drug’s paradoxical effect.

### 3.3. Cyclodextrin-Based Systems in Osteogenesis

#### 3.3.1. Hydroxyapatite/Cyclodextrin Composite Nanoparticles

Hydroxyapatite, the main mineral component of bone and tooth enamel, is frequently used to develop new dental repair materials. It is, however, a fragile material, requiring the formation of composites with controlled mechanical properties in order to be used in artificial implants. Cyclodextrins were reported to help design nanoparticles of hydroxyapatite with controlled particle size, good aggregation behaviour and superior osteogenic activity in vitro (when compared to pure hydroxyapatite particles) [61].

#### 3.3.2. Cyclodextrin–Bisphosphonate Conjugates

Bisphosphonates are approved as regulating agents for bone metabolic diseases. These drugs are able to bind to hydroxyapatite and inhibit bone resorption by osteoclasts, the bone cells responsible for cleaning up bone tissue to make room for new bone growth. When the action of osteoclasts is excessive, bone growth is unable to accompany their activity leading to loss of bone mass; the most well-known disease of this category is osteoporosis [62].

A covalently bonded conjugate of alendronate with β-CD (ALN-β-CD) was developed for hydroxyapatite targeting and in situ delivery of drugs that can be loaded into the host cavity, which remains free [63]. In a follow-up study, the conjugate ALN-β-CD was evaluated in vivo in bone defects of the mouse mandible, with histology results revealing that it can promote bone growth. Notably, the osteogenic effect of ALN-β-CD is very localised and centred on the injection site, whereas pure alendronate, in turn, results in new bone formation in a wide distribution, peripheral to the injection site [64]. This way, the osteotropic ALN-β-CD conjugate, primarily designed as a drug delivery carrier, can be used as a bone anabolic agent for the repair of focal bone defects.

**Figure 5:**
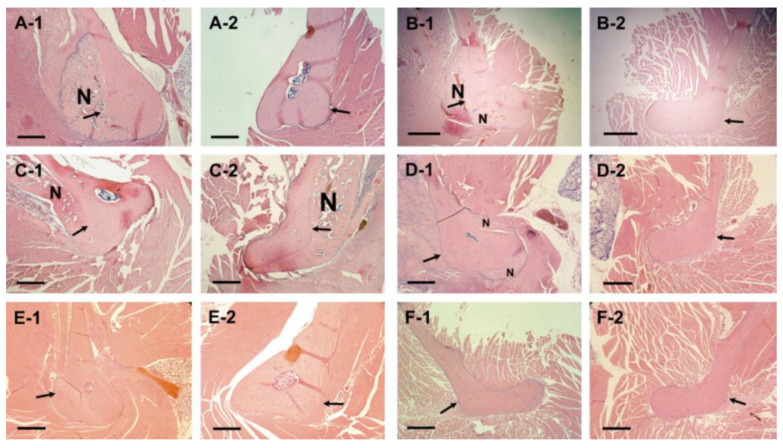
Histology of decalcified rat mandible pairs 24 days after treatments with different compounds. ALN-β-CD·prostaglandin E_1_ (**A-1**) vs. HPβCD·prostaglandin E_1_ (**A-2**); ALN-β-CD (**B-1**) vs. HPβCD (**B-2**); ALN-β-CD·prostaglandin E_1_ (**C-1**) vs. ALN-β-CD (**C-2**); ALN (**D-1**) vs. saline (**D-2**); prostaglandin E_1_ (**E-1**) vs. ethanol (**E-2**); saline (**F-1**) vs. no treatment (**F-2**). N = new bone. Bar = 0.5 mm. Arrow indicates the approximate site of injection. Reproduced with permission from Liu et al. [64]; copyright ©2007 Elsevier.

ALN-β-CD retains its originally planned drug delivery abilities, being thus suited for dual action. When the cyclodextrin cavity of ALN-β-CD was loaded with dexamethasone, a glucocorticoid often used to treat mucosal inflammation, the release was shown to occur in a very gradual way, taking more than 15 washout steps (in vitro, with saliva simulating fluid) to release 90% of the loaded drug [63]. Loading of ALN-β-CD with prostaglandin E_1_, a bone growth-promoting biomolecule, led to the gradual release, albeit somewhat faster than the one observed in the case of dexamethasone). Moreover, in vivo studies in a defect of the mouse mandible showed a very strongly localised bone anabolic reaction for the inclusion compound of ALN-β-CD·prostaglandin E_1_ (Figure 5), which resulted from the combined activity of its two components [64].

### 3.4. Cyclodextrin-Based Strategies for the Management of Periodontal Disease

#### 3.4.1. Materials Containing Cyclodextrins as Drug Carriers

The effect of a β-CD-containing gel on the efficacy of doxycycline for the management of periodontitis was evaluated by a randomised clinical trial on 33 human patients. The antibiotic was used in the form of a hydrogel at a concentration of 10%, either with or without β-CD, via topical application on the periodontal pocket. The gel containing 10% doxycycline and β-CD gel showed a significant improvement in periodontal clinical parameters (higher attachment levels and lower bleeding) as well as a reduction of plaque formation [65].

In another approach, the inclusion compound of β-CD with chlorhexidine was loaded into bacterial cellulose membranes aiming at periodontitis treatment. The membranes with the inclusion compound showed superior antimicrobial activity in vitro when compared with those loaded with only the pure drug [66]. The in vivo clinical efficacy of these antimicrobial-loaded membranes was not determined, thus warranting future studies.

#### 3.4.2. Cyclodextrins as Building Blocks for Innovative Materials with Sustained Release

Molecules of γ-CD can self-assemble into tridimensionally organised frameworks that are held together by interactions with cations such as Na^+^, K^+^ and even Fe^3+^. These structures are called γ-cyclodextrin metal–organic frameworks (γ-CD-MOFs) and they have the advantage of presenting a very high surface-to-volume area because of the large pores and interlinking channels that result from combining the cavities of various cyclodextrin molecules. Their quick dispersion in an aqueous medium, however, implies that the structure must be stabilised by a cross-linking agent in order to be suited for the prolonged delivery of active ingredients in the oral cavity [67].

**Figure 6:**
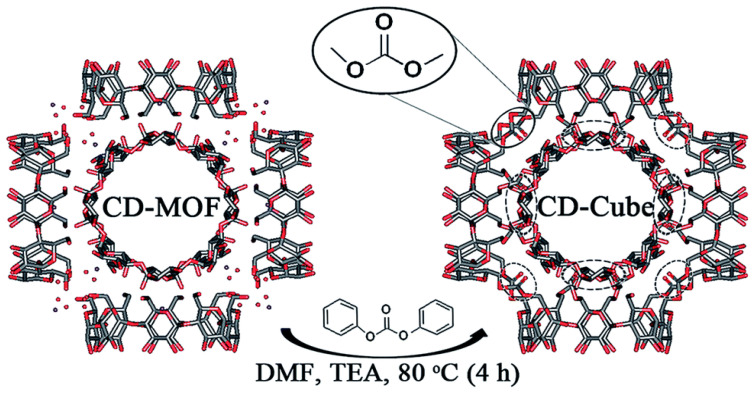
Reaction conditions and structural representation of the formation of a cross-linked cubic gel starting from γ-CD-MOF, depicted for one of its (γ-CD)_6_ cubic units and highlighting the kind of chemical links formed. Reproduced from Singh et al. [68] under a Creative Commons Attribution 3.0 Unported Licence (2017).

In a recent report, diphenyl carbonate was used as the crosslinking agent for γ-CD-MOFs; furthermore, triethylamine (TEA) was used as a catalyst, allowing the crosslinking reaction to be completed in as little as 4 h (Figure 6) [68]. The resulting particles retained the cubic shape of the original γ-CD-MOFs particles that formed them, but were insoluble in water and able to carry a high load of actives.

In a subsequent study, the particles were loaded with iodine by immersion into a solution of KI_3_ for 2 h, which afforded a load of c.a. 30% (*w*/*w*) [69]. The release profile of iodine from the loaded particles was determined in vitro using artificial saliva. Half of the load in iodine was released in the first 12 h of immersion, followed by a period with practically no further iodine release. Following this, the therapeutic effect in vivo was evaluated in a rat periodontitis model, comparing the iodine-loaded gel particles with minocycline (a model drug). Both the gel and minocycline were administered every three days for a total period of four weeks, and both showed similar results in the symptomatic relief of periodontitis. Histological examination of the periodontium confirmed these results: in contrast with non-treated rats, which exhibited a large number of infiltrating inflammatory cells and osteolytic osteoclast cells into the bone tissue, the tissue of rats treated with iodine-loaded gel or with a minocycline ointment revealed a low number of infiltrated inflammatory cells, meaning reduced tissue inflammation. In addition, the connective tissue regained its normal healthy appearance, with no osteoclasts in the periosteum and osteoblasts being observed instead. These histological changes demonstrate that a four-week treatment with either minocycline or iodine-loaded gel restores periodontal gingival tissue to a healthy morphological condition.

## 4. Outlook and Future Perspectives

The present paper provides an overview of the multi-faceted applications of cyclodextrins in dentistry, focusing on two main aspects of oral health: prevention and treatment. The relevance of these molecules is associated with the plethora of benefits they can bring to a formulation and also with their safety. Cyclodextrins are generally considered safe for use in oral care products, as they are biocompatible, non-toxic, and non-irritating.

In oral care and health maintenance, the use of cyclodextrins is shown to extend far beyond their classical solubilising and stabilising role for antimicrobial agents, flavourants and desensitising agents. While solubilisation is important and it remains one of the most widely used applications, owing to the ability of cyclodextrins to form inclusion compounds with hydrophobic guests, to protect them from degradation, to increase their aqueous solubility and, often, also their efficacy and bioavailability, cyclodextrins can further act as delivery systems, controlling the release of included active ingredients and carrying them to targeted areas in the oral cavity. In other instances, cyclodextrins are employed as formulation helpers, transforming liquid ingredients into powders that are easier to manipulate, store and combine with other ingredients (e.g., by avoiding undesired interactions in a formulation). Furthermore, cyclodextrins have been studied for their potential to remove malodorous compounds, including volatile sulfur compounds responsible for bad breath. Herein, an example is shown of a cyclodextrin-based oral care product developed to capture and eliminate such sulphur compounds in the mouth, providing long-lasting fresh breath.

A growing trend in oral care is the development of natural compositions, in which cyclodextrins have several relevant applications and pose as key ingredients because they are regarded as natural ingredients themselves. Cyclodextrins can modulate the taste and texture of products containing natural extracts, either by masking bitterness or by enhancing the organoleptic properties of odour and flavour components. This improves the sensory experience of using these products and makes them more pleasant to use. Regarding bioactive antiseptic agents, cyclodextrins were shown to play a determinant role in contributing to the anti-bacterial and anti-viral properties or oral compositions.

Cyclodextrins were also shown to play a diversity of roles in dental pastes and cements. These roles can vary depending on the specific type and application of the dental product. Innovative binding agents are created from cyclodextrin-appended methacrylate monomers, giving rise to resins with high binding ability in tandem with drug-carrying properties. Similarly, modulation of binding polymers by threading some of their side chains into cyclodextrins is reported to afford more shear-resisting materials. In opposition, cyclodextrins can also tailor the binders into light-activated degradation in the case of materials that are used in temporary repairs and fixtures and that require easy removal when they are no longer needed.

New cyclodextrin-derived molecules for dental applications were also highlighted in this review, with two main categories: a bone-targeting class of bisphosphonate-cyclodextrin hybrids, able to promote bone growth either by themselves or with the help of osteogenic drugs carried inside the cavity of the cyclodextrin, and cyclodextrin cubic gel particles, designed to have an ultra-high loading capacity as they are made using the highly porous γ-CD-MOFs as template.

The vast and diverse panorama of cyclodextrin applications in dentistry that is described in the present review serves to demonstrate the interest and versatility of these molecules. As research in this field continues, it is likely that cyclodextrins will find even more applications in the development of advanced oral care products. However, the current panorama also shows a dichotomy in the use of cyclodextrins in dentistry. Indeed, only in simple oral healthcare products such as mouthwashes and toothpastes have cyclodextrins been able to break the barriers of transition from bench to market. In turn, their use in materials for dental repair and treatment of disorders in the oral cavity remains in the research phase. A common trait of these studies is the lack or little amount of data on biocompatibility. This is particularly evident in regard to investigation with clinical and pre-clinical models (very few papers available). These studies are, nonetheless, paramount in future research as it is vital to ensure that the new cyclodextrin-based materials and new cyclodextrin molecules are safe to use in human biomedical applications.

## Figures and Tables

**Table 1:** Pharmaceutical products with native and chemically modified cyclodextrins.

Cyclodextrin	Pharmaceutical Dosage Forms				
	Oral	Nasal	Ocular	Dermal	Parenteral
α-CD	—	—	—	—	✓
β-CD	✓	—	✓	✓	not allowed
γ-CD	✓	—	—	✓	—
HPβCD	✓	—	✓	✓	✓
RAMEB	not allowed	✓	✓		not allowed

The check sign (✓) denotes known cases of dosage forms containing CDs for a particular delivery route; adapted from ref. [19].
